# First Cytogenetic Analysis of *Hemidactylus mercatorius* Gray, 1842 Provides Insights on Interspecific Chromosomal Diversification in the Genus *Hemidactylus* (Squamata: Gekkonidae)

**DOI:** 10.3390/life14020181

**Published:** 2024-01-25

**Authors:** Marcello Mezzasalma

**Affiliations:** Department of Biology, Ecology and Earth Sciences, University of Calabria, Via P. Bucci 4/B, 87036 Rende, Italy; marcello.mezzasalma@unical.it

**Keywords:** chromosomes, evolution, gecko, karyotype, NORs, C-banding, reptiles

## Abstract

This contribution provides the first karyotype description of *Hemidactylus mercatorius* and discusses the interspecific chromosome diversification in the genus. Chromosomal analysis was performed on samples from different Malagasy populations using standard karyotyping, Ag-NOR staining, and banding methods (sequential C-banding + Giemsa, + Chromomycin A_3_, +4′,6-diamidino-2-phenylindole). Irrespective of sex or sampling locality, *H. mercatorius* shows a karyotype of 2n = 42 with metacentric (1, 18–21), submetacentric (4), subtelocentric (5, 11), and acrocentric pairs (all the remaining pairs). There was no heteromorphic chromosome pair and no clear distinction between macro- and microchromosomes. NORs were localised close to the centromeres of a medium acrocentric pair (14). Heterochromatic blocks were identified on the telomeric and centromeric regions of most chromosome pairs. A comparison with the karyotype of *H. mabouia* highlights that the different morphology of several chromosome pairs clearly distinguishes the two species, contrasting the previously proposed synonymy. The differences between the karyotypes of *H. mercatorius* and *H. mabouia* concern the number of biarmed and acrocentric elements, suggesting the occurrence of several chromosome inversions. Considering all the available karyotype data on *Hemidactylus* and its sister genus *Cyrtodactylus*, it is possible to advance an evolutionary hypothesis on their chromosomal evolution, starting from a common ancestor with 2n = 48 and all acrocentric elements. From this ancestral condition, the karyotype diversification in the two genera has been prevalently characterised by a progressive accumulation of fusions and inversions which have reduced the total chromosome count and increased the number of biarmed chromosomes.

## 1. Introduction

The peculiar evolutionary history of the biodiversity of Madagascar has been driven by a unique combination of geographical isolation, high environmental variety, and complex biogeographic connections (in the form of Gondwanan vicariance and Cenozoic dispersal) with mainland Africa, Asia (mainly with the Indian subcontinent), and South America [1,2]. These factors together contribute to defining Madagascar as a classic model region for studying evolutionary processes at different taxonomic levels [2,3].

Madagascar hosts more than 450 reptile species, and it is ranked among the countries with the highest herpetological diversity [3,4]. However, the highly endemic squamate fauna of Madagascar is still relatively poorly understood, despite the progress achieved during the last decades with several new species described every year [4].

To date, six families of snakes (Elapidae, Psammophiidae, Boidae, Pseudoxyrhophiidae, Typhlopidae, and Xenotyphlopidae) and six families of lizards (Agamidae, Chamaeleonidae, Opluridae, Gekkonidae, Gerrhosauridae, and Scincidae) are known to be present in Madagascar. Among them, the Malagasy Gekkonidae comprise 11 different genera (*Blaesodactylus*, *Matoatoa*, *Ebenavia*, *Geckolepis*, *Gehyra*, *Lygodactylus*, *Hemidactylus*, *Paroedura*, *Paragehyra*, *Phelsuma*, and *Uroplatus*) and more than 140 species currently described [3,4].

Three gecko species of the genus *Hemidactylus* are currently known to contribute to the reptile diversity of the island: *Hemidactylus mercatorius* Gray, 1842; *H. frenatus* Duméril & Bibron, 1836; and *H. platycephalus* Peters, 1854 (The Reptile Database, 2023) [4]. These species can be found in syntopy in different regions, but among them, only *H. mercatorius* is considered a native species in Madagascar [5,6,7]. *Hemidactylus mercatorius* is also present in Aldabra (Seychelles), while its populations in the main island group of the Seychelles, Mayotte, São Tomé and Príncipe, the Comoro islands, and Equatorial Guinea are considered to be of uncertain origin [4,6,7]. *Hemidactylus frenatus* is a virtually ubiquitous lizard in tropical and subtropical regions. This species is native to South and Southeast Asia and the Indo-Australian Archipelago, but historical human-mediated colonisation is thought to be responsible for the presence of the species in many of the islands of the South Pacific, Hawaii, Central and South America, the United States, East Africa, and West Madagascar [4,6,8,9,10]. In turn, *H. platycephalus* naturally occurs in Central and East Africa (Somalia, Ethiopia, Kenya, Mozambique, Tanzania, Zambia, Zimbabwe, and Malawi), Mayotte, and Anjouan but has also been historically introduced in other Comoro islands and Madagascar [4,7].

The taxonomic status of *H. mercatorius* has been highly debated in the last decades. The species was synonymised with *H. mabouia* [11] but later reconsidered a full species (Kluge, 2001) [12]. *Hemidactylus mercatorius*, *H. mabouia*, *H. frenatus*, and *H. platycephalus* have been the targets of several molecular studies [5,6,7,8,13,14]. These studies greatly improved our understanding of the phylogenetic relationships and historical biogeography of different populations, but they also highlighted the occurrence of species complexes whose taxonomy should be better assessed. On the other hand, cytogenetic methods have been applied only to *H*. *frenatus* and *H. mabouia*, which showed a karyotype composed of 2n = 40 and 42 chromosomes, respectively [10,15,16,17,18,19].

Cytogenetic analyses, especially when associated with molecular sequences, may provide important taxonomic and evolutionary information on the taxa studied. In fact, different chromosomal configurations can represent ancestral or apomorphic states which can be useful to understand evolutionary dynamics (see e.g., [10,20,21]. Furthermore, squamate reptiles represent particularly interesting model organisms in evolutionary cytogenetics. In fact, they display a high variability in chromosome number, morphology, and the number and location of different chromosomal markers [22]. Sex chromosome systems are also highly variable in squamates and particularly in lizards. Many families and genera include simple and multiple sex-chromosome systems, which can emerge from non-homologous macro- and microchromosome pairs in distinct evolutionary lineages, covering all the different hypothesised steps of diversification of heterogametic sex chromosome pairs from homomorphic to heteromorphic and completely heterochromatic chromosomes [23].

This paper shows the results of the first karyological study, performed with different staining and banding methods, on various individuals from two different Malagasy sampling locations of *H. mercatorius*. The results obtained were compared with the available literature data on *Hemidactylus* and its sister genus *Cyrtodactylus* [10,15,16,18,19,24,25]. The available karyological data were then superimposed on the existing phylogenies of *Hemidactylus* and *Cyrtodactlylus* (Pyron et al., 2013; Rato et al., 2021) [14,26], thus providing new insights into their chromosomal diversification.

## 2. Materials and Methods

### 2.1. Sampling

This study is based on archival cell suspensions stored at −20 °C and dating back to field activities carried out between 2002 and 2003. Six individuals of *H. mercatorius* from Madagascar were analysed in this study. The samples were collected during fieldwork in 2002–2003 by various researchers, and no animal was sampled during the realisation of this study. Taxonomic attribution, field number, sex, and sampling location of all the samples analysed in this study are provided in Table 1. After capture, animals were injected with a 0.5 mg/mL colchicine solution (0.1 mL/10 g body weight). Tissue samples (intestine, spleen, and gonads) were incubated for 30 min in hypotonic solution (KCl 0.075 M + sodium citrate 0.5%, 1:1), fixed, and conserved in Carnoy’s solution (methanol and acetic acid, 3:1). The fixed material was temporarily preserved at 4°C and transferred to the laboratory where it was stored at −20 °C and subsequently processed as described below. The taxonomic attribution of the study samples was determined by means of a preliminary molecular analysis using a trait of the 12S rDNA (samples GA 507–510) (see below) or following the taxonomic attribution by Cocca et al. [27] (samples FAZC 11897 and FAZC 11898) based on morphological and/or molecular analyses.

### 2.2. Molecular Analysis

A preliminary molecular analysis was realised to assess the taxonomic status of the samples studied and associate DNA sequences with the newly described karyotypes. DNA was extracted from tissue samples and cell suspensions according to Sambrook et al. (1989) [28]. A fragment of the mitochondrial 12S rRNA gene (12S) of about 400 bp was amplified following Kocher et al. [29] using the primer pair 12Sa 5′-AAACTGGGATTAGATACCCCACTAT-3′ and 12Sb 5′-GAGGGTGACGGGCGGTGTGT-3′. This marker was chosen considering its wide use on geckos of the genus *Hemidactylus* and the number of available sequences on GenBank [7,14,30].

PCR was conducted in a reaction volume of 25 μL using the following parameters: initial denaturation at 94 °C for 5 min, followed by 36 cycles at 94 °C for 30 s, 55 °C for 30 s, 72 °C for 45 s, and a final extension for 7 min at 72 °C. Amplicons were sequenced in both directions on an automated sequencer ABI 377 (Applied Biosystems, Foster City, CA, USA) using BigDye Terminator 3.1 (ABI) kit. The resulting electropherograms were manually checked, edited, and aligned with Clustal W using Chromas Lite 2.6.6 and BioEdit 7.2.6.1 [31]. For taxonomic attribution, the newly determined sequences were blasted in GenBank, aligned, and compared with available homologous traits used in previous phylogenetic and taxonomic studies on the genus *Hemidactylus* [7,14,30]. Sequences showing an identity score > 98% were considered conspecific.

### 2.3. Cytogenetic Analysis

The cytogenetic analysis was performed using the archival cell suspensions obtained as reported above, and metaphase chromosomes were obtained with the air-drying method, as described in Mezzasalma et al. [32]. Chromosomes were then stained with conventional colorations (5% Giemsa solution at pH 7), Ag-NOR staining [33], C-banding according to Sumner [34], and sequential C-banding + fluorochromes (CMA_3_ and DAPI) following Mezzasalma et al. [35]. Metaphase plates were detected and recorded using an optical and an epifluorescent microscope (Axioscope Zeiss, Oberkochen, Germany) equipped with an image analysis system. Karyotype reconstruction and the calculation of the chromosome relative length (RL = length of a chromosome/total karyotype length) and centromeric index (CI = (length of the short arm/total length of the chromosome) (see Table 2) were performed after scoring and recording at least 15 metaphase plates per sample studied, and chromosomes were identified as metacentric (m), submetacentric (sm), subtelocentric (st), and acrocentric (a) following the traditional classification proposed by Levan et al. (1964) [36].

## 3. Results

### 3.1. Molecular Analysis

The selected fragment of the 12S (of about 400 bp) was successfully amplified in all the individuals studied, with the exception of the samples FAZC 11897 and FAZC 11898, which have been already taxonomically identified in Cocca et al. [27]. The preliminary molecular analysis facilitated a taxonomic assessment of the samples studied as reported in Table 1. The maximum identity scores (>99% with a specimen from Isalo, Madagascar, Accession number: MW665156) retrieved between the samples analysed in this work and homologous sequences of *H. mercatorius* deposited in GenBank used in previous molecular studies provided a reliable taxonomic attribution, which is shown in Table 1. The newly generated DNA sequences were deposited in GenBank under the Accession numbers: PP001486-PP001489.

### 3.2. Cytogenetic Analysis

**Figure 1:**
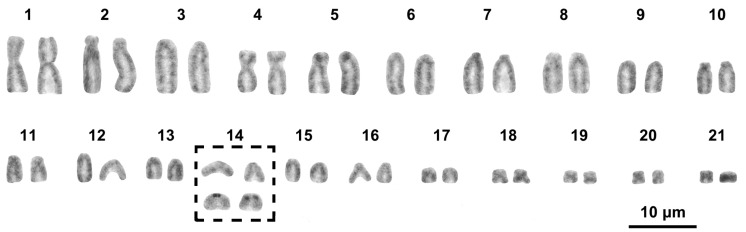
Karyotype of *H. mercatorius* stained with Giemsa. The NOR-bearing elements of pair 14 (in the bracket) are stained with Giemsa (up) and Ag-NOR staining (down).

**Figure 2:**
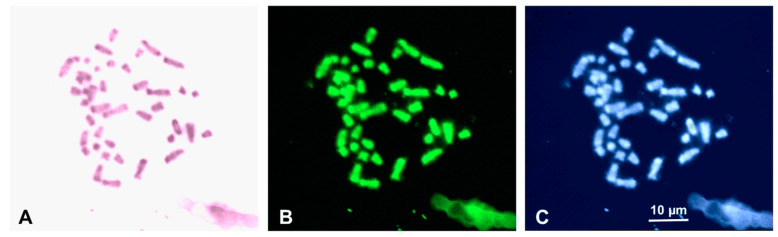
Metaphase plate of *H. mercatorius* sequentially stained with C-banding + Giemsa (**A**) + CMA (**B**) and + DAPI (**C**). Scale bar applies to all images.

All the studied individuals of *H*. *mercatorius* (n = 6, see Table 1) showed a karyotype composed of 2n = 42 chromosomes, with metacentric (pairs 1, 18–21), submetacentric (pair 4), subtelocentric (pairs 5, 11), and acrocentric elements (all the remaining pairs) (Fundamental number, total arm number, FN = 54) (Figure 1; Table 2). There was no occurrence of any heteromorphic chromosome pair and no evident distinction between macro- and microchromosome pairs (Figure 1). The Ag-NOR staining localised loci of NORs close to the centromeres of the chromosomes of a medium acrocentric pair, here tentatively identified as pair 14 because of its relative size (Figure 1, Table 2). After sequential C-banding (Figure 2), heterochromatic blocks were identified on the telomeric and centromeric regions of most chromosome pairs (including the NOR-bearing chromosome pair 14), independently of the sex or sampling locality of the individuals studied. In general, chromosomal heterochromatic content was evident after either C-banding + Giemsa or C-banding + fluorochromes. No unpaired or largely heterochromatic chromosome was detected in the karyotype of *H. mercatorius* after sequential C-banding (Figure 2).

## 4. Discussion

**Figure 3:**
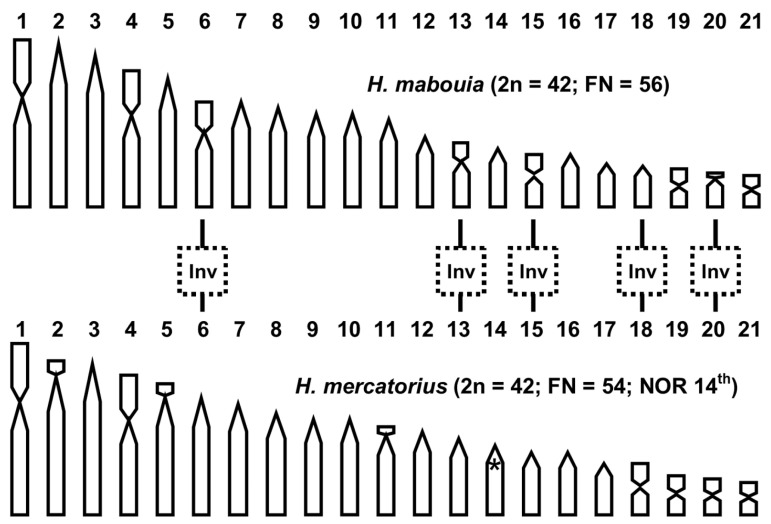
Haploid karyograms of *H. mercatorius* (this study) and *H. mabouia* [24,25] with the hypothesised transitional chromosomal rearrangements. * = loci of NORs. Inv = chromosome inversion.

Several molecular analyses on Malagasy populations of *H. mercatorius* showed the existence of limited genetic variability within the island, but distinct haplotypes occur in different regions (see [8,27,37]). The results of the present study show that the individuals of *H. mercatorius* here considered are karyologically uniform in the chromosome number and morphology, localisation of NORs clusters, and general chromosomal content and distribution of heterochromatin. However, interestingly, the results here presented support the specific status of *H. mercatorius* and its distinctiveness from *H. mabouia*, in contrast to the previously proposed synonymy between the two species [11]. In fact, the chromosomes of *H. mabouia* have been described from samples from different populations by Beçak et al. [24] and McBee et al. [25], and these showed the same karyotype formula of 2n = 42 chromosomes with biarmed (pairs 1, 4, 6, 13, 15, 19, and 21) and acrocentric pairs (all the remaining pairs). This chromosome formula can be considered typical of the species because it characterises different molecular clades of *H. mabouia* with a wide geographical distribution (see [7]). The comparison between the karyotypes of *H. mercatorius* and *H. mabouia* (see Figure 3) shows the same chromosome number of 2n = 42 but a different morphology of several chromosome pairs (6, 13, 15, 18, and 20), which are all acrocentric in *H. mercatorius* (this study) and biarmed in *H. maboiua* [24,25].

Chromosome inversions are the most likely rearrangements involved in shaping the different morphology of those chromosome pairs as they have been described in different gecko lineages and may often occur among sister lineages [18,38], but centromere repositioning cannot be excluded as an alternative hypothesis [39]. These chromosome rearrangements might have occurred either before or after the molecular diversification between *H. mercatorius* and *H. maboiua*, but they currently appear to be fixed chromosome characters of cytotaxonomic relevance. In fact, chromosomal inversions are well known to potentially generate and/or reinforce genetic isolation by establishing postzygotic barriers reducing the fertility of chromosomal heterozygotes [20]. Moreover, the relatively high number of chromosome changes identified among *H. mercatorius* and *H. mabouia* appear to be of particular interest considering their sister-clade status and suggest a rapid karyotype diversification.

**Figure 4:**
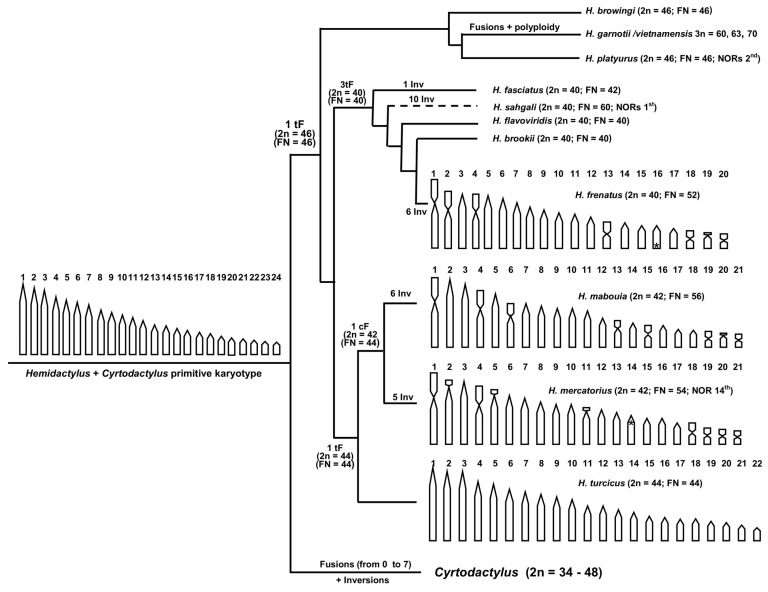
Phylogenetic relationships of *Hemidactylus* species with a known karyotype (tree topology redrawn from Pinho et al. and Pyron et al.) [7,26]. Superimposed with karyotype data of *H. mercatorius* (present paper), *H. mabouia* [24], *H. frenatus*, and *Cyrtodactylus* [43,44]. cF = centric Fusion; tF = tandem fusion; Inv = inversion. * = loci of NORs.

To better understand the general intra- and intergeneric chromosome evolution, the possible polarity of the chromosome rearrangements involved, and the relative increase or decrease in the number of biarmed elements, it is useful to extend the karyological comparisons to other species of the genus *Hemidactylus*, also taking into consideration their phylogenetic relationships and the known karyotypes of the genus *Cyrtodactylus*, the sister clade to *Hemidactylus* [7,26] (see Figure 4). In particular, the known karyological variability in *Hemidactylus* ranges from 2n = 40 (in *H. fasciatus*, *H. flavoviridis*, *H. brookii*, and *H. frenatus*) [16,40,41,42] to 2n = 44 (in *H. turcicus*) and 2n = 46 (in *H. bowringii* and *H. platyurus*) [18] (Figure 4). Polyploidy is also known to occur in the genus in the form triploidy in the *H. garnotii*/*vietnamensis* species complex [41]. In turn, chromosome data on the genus *Cyrtodactylus* are available from seven species, with karyotypes ranging from 2n = 34 (with many meta- and submetacentric elements) to 2n = 48 (and all acrocentric elements) [43,44] (see Figure 4).

In the family Gekkonidae, chromosomal diversification has been hypothesised to have possibly occurred either by an augmentation or by a reduction in the total number of chromosomes. In particular, King [45] identified several possible ancestral karyotypes in the subfamily Gekkoninae (from 2n = 32 to 2n = 46), suggesting that different evolutionary lineages went through a distinct combination of chromosome rearrangements (fusions and inversions), mostly toward a reduction of the total chromosome number. More recently, Trifonov et al. [18] proposed a karyotype of 2n = 40 with all acrocentric chromosomes as the primitive condition in *Hemidactylus* and hypothesised that successive events of diversification occurred mostly toward an increase in the number of chromosomes by chromosome fission up to 2n = 46. However, it is possible to advance an alternative evolutionary hypothesis based on the most parsimonious number of chromosome rearrangements, starting from the common ancestor of *Hemidactylus* and *Cyrtodactylus* with a putative karyotype composed of 2n = 48 with all acrocentric chromosomes (Figure 4). In *Cyrtodactylus* (2n = 34–48), this hypothesised primitive condition was either conserved or modified through a progressive number of chromosome fusions which reduced the chromosome number to 2n = 34, increasing the count of biarmed elements (Figure 4). Similarly, a single chromosome fusion likely reduced the chromosome number of the *Hemidactylus* common ancestor to 2n = 46 (producing a similar karyotype to those of *H. bowringii* and *H. platyurus*), while additional chromosome fusions and inversions led to the formation of karyotypes with a lower total chromosome number (2n = 44–40) but a higher number of biarmed elements (Figure 4). This evolutionary scenario is more similar to that initially proposed by King [45] and seems to be supported by other evidence on squamates. In fact, a higher total chromosome number and higher ratio of acrocentric elements are considered primitive characters in the karyotype of most squamate taxa (see e.g., [46,47,48]). Moreover, similar tendencies toward a general reduction in the total chromosome number and an increase in the number of biarmed elements by means of chromosome fusions have been observed in several taxa of the family Gekkonidae such as *Blaesodactylus*, *Lygodactylus*, *Paroedura*, *Uroplatus*, and several circum-Indian Ocean leaf-toed geckos [32,48,49,50], as well as in other squamate families [51,52]. Multiple observations in different taxonomic groups of geckos of similar, independent occurrences of a reduction in the total number of chromosomes by means of chromosome fusions and inversions suggest the possible occurrence of a convergent karyotype evolution. However, this hypothesis, which has previously been suggested also for other squamate taxa (see e.g., [23,47,51] and references therein), should be confirmed with additional experimental analyses.

Concerning the chromosomal localisation of the loci of NORs in *Hemidactylus*, experimental data are available only for *H. mercatorius* (on the 14th pair) (present paper), *H*. *frenatus* (on the 16th pair), and *H*. *platyurus* (on the 2nd pair) [17,43]. These data evidence the variability of the NOR-bearing pair, but they are also currently too limited to understand the possible diversification pathways of these chromosome markers in the genus.

Sequential C-banding (+Giemsa, +DAPI, +CMA_3_) did not evidence the presence of differentiated sex chromosomes in *H. mercatorius*, and similarly to what is usually observed in squamates, heterochromatic blocks are mostly evident on the centromeric and telomeric regions [23,53]. To date, the only evidence of differentiated sex chromosomes in *Hemidactylus* is represented by a possible ZZ/ZW sex chromosome system in *H. platyurus* [18]. In the phylogenetically closely related *Cyrtodactylus*, Keating et al. [54] identified putative sex-determination systems with either male or female heterogamety in different species (XX/XY in *C*. *chaunghanakwaensis* and ZZ/ZW in *C*. *pharbaungensis*) using RAD-seq methods. This evidence suggests the occurrence of a sex chromosome turnover in the genus and further supports the plasticity of sex-determination systems in Gekkonidae (see e.g., [23,55,56]).

The current main limitation of a deeper understanding of the karyotype and sex chromosome evolution in *Hemidactylus* and its sister genus *Cyrtodactylus* is represented by the relatively low number of species with known karyotypes. This is not surprising considering that despite their high chromosomal variability, only a fraction of the formally described squamate species have a known karyotype [23]. Additional karyotype descriptions and cytogenetic analysis using a combination of traditional and molecular cytogenetics should be performed on a higher number of species in order to thoughtfully describe the chromosome variability of *Hemidactylus* and *Cyrtodactylus*, uncover the origin and diversification of sex-determination systems, and provide further support to the hypothesised pathways of their karyotype evolution.

## 5. Conclusions

Chromosome mutations can be useful taxonomic and evolutionary markers in comparative cytogenetic studies. At low taxonomic levels, they can be coupled with molecular data and used for difficult diagnoses as well as in order to achieve a better understanding of the evolution of closely related taxa. At higher taxonomic levels, the progressive accumulation of chromosome changes can be used to identify the particular evolutionary pathways that characterise the karyotype diversification of the studied taxa. This contribution presents the first karyotype description of *H. mercatorius* and a comparison with the chromosome complement of the evolutionary closely related *H. mabouia*. These two species, which were previously considered synonyms, show the same chromosome number (of 2n = 42) but a different morphology for several chromosome pairs (6, 13, 15, 18, and 20), which are all acrocentric in *H. mercatorius* and biarmed in *H. maboiua*. Chromosomal inversions and/or centromeric shifts are the most likely chromosome rearrangements involved in the karyological diversification of the two species. Furthermore, taking into consideration the available karyotypes of *Hemidactylus* and its sister genus *Cyrtodactylus*, it is possible to advance an evolutionary hypothesis based on the most parsimonious number of chromosome rearrangements, starting with a putative common ancestor with a karyotype of 2n = 48 with all telocentric elements. Following this hypothesis, the karyological diversification in the genus *Hemidactylus* likely proceeded toward a reduction in the total chromosome number and an augmentation in biarmed chromosomes through a progressive accumulation of chromosome fusion and inversions. It should also be noted that similar tendencies toward a general reduction in the total chromosome number and an increase in the number of biarmed elements by means of chromosome fusions have been previously described in several other genera of the family Gekkonidae (such as *Blaesodactylus*, *Lygodactylus*, *Paroedura*, and *Uroplatus*), as well as in other families of squamates.

## Figures and Tables

**Table 1:** Origin and sex of the studied samples of *H*. *mercatorius*. * = taxonomic attribution following Cocca et al. [27].

Specimen	Locality	Sex
GA 507	Mandrivazo	male
GA 508	Mandrivazo	female
GA 509	Mandrivazo	male
GA 510	Mandrivazo	male
FAZC 11897 *	Analalava forest	juvenile
FAZC 11898 *	Analalava forest	male

**Table 2:** Chromosome relative length (RL) and centromeric index (CI) of chromosomes of *H. mercatorius*. Cp = chromosome pair, M = metacentric, sM = submetacentric, sT = subtelocentric, T = acrocentric.

Cp	RL	CI
1	9.0 ± 1.1	43.5 ± 3.7 (M)
2	8.9 ± 1.0	10.6 ± 1.7 (T)
3	8.3 ± 0.9	4.2 ± 2.0 (T)
4	7.6 ± 0.8	34.1 ± 2.5 (sM)
5	7.0 ± 0.7	15.6 ± 3.0 (sT)
6	6.1 ± 0.7	3.3 ± 1.1 (T)
7	6.0 ± 0.4	5.2 ± 2.1 (T)
8	6.0 ± 0.5	4.8 ± 3.0 (T)
9	5.5 ± 0.6	6.3 ± 2.9 (T)
10	4.9 ± 0.6	2.8 ± 1.2 (T)
11	4.1 ± 0.5	15.8 ± 3.5 (sT)
12	3.9 ± 0.4	4.8 ± 2.8 (T)
13	3.4 ± 0.4	9.3 ± 3.7 (T)
14	3.3 ± 0.5	7.4 ± 4.2 (T)
15	2.7 ± 0.3	6.5 ± 3.0 (T)
16	2.6 ± 0.7	10.2 ± 1.9 (T)
17	2.6 ± 0.6	3.2 ± 2.1 (T)
18	2.5 ± 0.4	42.8 ± 4.2 (M)
19	2.1 ± 0.5	44.4 ± 2.7 (M)
20	1.9 ± 0.5	42.9 ± 3.8 (M)
21	1.8 ± 0.4	45.3 ± 4.0 (M)

## Data Availability

All the chromosome data are available within this manuscript. The newly generated DNA sequences were deposited in GenBank under the Accession numbers: PP001486-PP001489.
